# Diagnostic value of intraoperative ultrasonography in assessing thoracic recurrent laryngeal nerve lymph nodes in patients with esophageal cancer

**DOI:** 10.1186/s12885-018-4643-8

**Published:** 2018-07-13

**Authors:** Jianwei Wang, Min Liu, Jingxian Shen, Haichao Ouyang, Xiuying Xie, Ting Lin, Anhua Li, Hong Yang

**Affiliations:** 1 Sun Yat-sen University Cancer Center, State Key Laboratory of Oncology in South China, Collaborative Innovation Center for Cancer Medicine, 651 Dong Feng Road East, Guangzhou, 510060 Guangdong China; 2 Guangdong Esophageal Cancer Institute, Guangzhou, China; 3 Shenzhen Seventh People’s Hospital, Shenzhen, 518000 China

**Keywords:** Esophageal cancer, Intraoperative ultrasonography, Recurrent laryngeal nerve nodal metastases

## Abstract

**Backgroud:**

The incidence of recurrent laryngeal nerve (RLN) injury has increased due to RLN lymph node dissection. The aim of this study was to evaluate the ability of intraoperative ultrasonography (IU) to detect RLN nodal metastases in esophageal cancer patients.

**Methods:**

Sixty patients with esophageal cancer underwent IU, computed tomography (CT), and endoscopic ultrasonography (EUS) to assess for RLN nodal metastasis. Sensitivity, specificity, positive predictive value (PPV), and negative predictive value (NPV) were compared.

**Results:**

The sensitivities of IU, CT, and EUS in diagnosing right RLN nodal metastases were 71.4, 14.3, and 30.0%, respectively, and a significant difference among these three examinations was observed (χ2 = 10.077, *P* = .006). The specificities of IU, CT, and EUS for diagnosing right RLN nodal metastasis were 67.4, 97.8, and 95.0%, respectively, and a significant difference was observed (χ2 = 21.725, *P* < .001). No significant differences in either PPV or NPV were observed when diagnosing right RLN nodal metastases. For diagnosis of left RLN lymph nodal metastases, the sensitivities of IU, CT, and EUS were 91.7, 16.7, and 40.0% respectively. There was a significant difference among these diagnostic sensitivities (χ2 = 14.067, *P* = .001). The specificities of IU, CT, and EUS for diagnosis of left RLN nodal metastases were 79.2, 100, and 82.5%, respectively and a significant difference was observed (χ2 = 10.819, *P* = .004). No significant differences were observed in PPV or NPV for these examinations when diagnosing left RLN nodal metastases.

**Conclusion:**

Intraoperative ultrasonography showed superior sensitivity compared with preoperative CT or EUS in detecting RLN lymph node metastasis in patients with thoracic esophageal cancer.

## Background

Esophageal cancer is one of the most common cancers in the world, with more than 455,800 new cases and 400,200 deaths occurring annually worldwide [1]. In China, over 90% of all cases of esophageal cancer are secondary to squamous cell carcinoma. Esophageal squamous cell carcinoma often metastasizes to thoracic recurrent laryngeal nerve (RLN) lymph nodes. Therefore, determining whether or not RLN lymph nodes are involved is important in assessing the spread of the cancer.

RLN lymph node dissection plays an important role in the treatment of esophageal cancer. RLN nodal dissection can provide accurate staging, achieve R0 resection, and improve prognosis. Up to now, however, there has been no reliable procedure other than systematic RLN lymphadenectomy for assessment, despite the fact that routine lymphadenectomy increases surgical morbidity [2–4] due to recurrent laryngeal nerve injury.

In a preliminary report [5], it was suggested that intraoperative ultrasonography (IU) was safe and feasible when used for the detection of RLN lymph nodes metastases in patients with thoracic esophageal cancer. The purpose of this study was to evaluate the ability of IU to detect RLN nodal metastases in esophageal cancer patients. A secondary aim was to compare the effectiveness of IU with preoperative computed tomography (CT) and endoscopic ultrasonography (EUS).

## Methods

### Patients

The Institutional Review Board of Clinical Research approved this study. All patients provided their written informed consent prior to enrolling in the study.

### Eligibility criteria

Eligible patients had histologically confirmed, potentially resectable thoracic esophageal squamous cell carcinoma; aged 18 to 70 years; suitable for McKeown esophagectomy including two-field lymphadenectomy with total mediastinal lymph node dissection [3]; had adequate hematological function with white blood cell ≥4·0 × 10^9^/L, neutrophil ≥1·5 × 10^9^/L, platelet ≥100·0 × 10^9^/L and hemoglobin ≥90 g/L; with normal renal and hepatic function; had a Karnofsky performance score (KPS) of 90 or better; and able to sign informed consent.

All the esophagectomies were performed by the same surgeon. All patients received chest and upper abdominal CT plain and contrast enhanced, esophagogastroduodenoscopy with ultrasound endoscopy preoperatively. The 7th edition of the International Union Against Cancer and American Joint Committee on Cancer staging system for Esophagus and Esophagogastric Junction was used [4].

## Materials

We used an ultrasound system produced by Aloka Co. Ltd., Japan. And a laparoscopic ultrasound probe (UST-5536- 7.5 MHz) was applied in this study. The probe had a frequency range of 5–10 MHz and the dimensions of it was 10 mm in diameter and 38 mm in length.

### IU examination

**Fig. 1:**
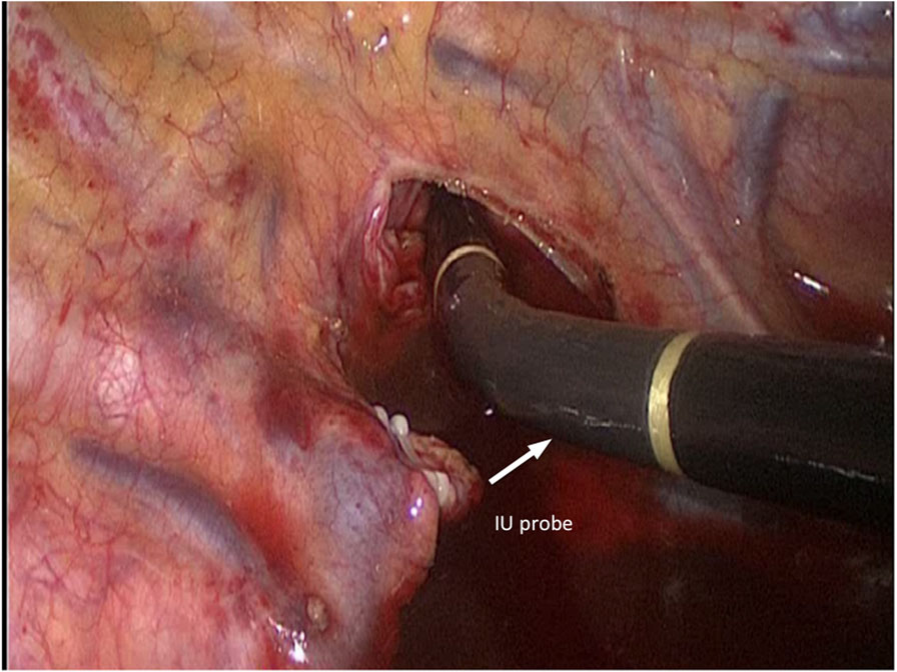
The probe of intraoperative ultrasonography (IU) directly scanned the recurrent laryngeal nerve (RLN) region

During the McKeown esophagectomy, the surgeon mobilized the thoracic esophagus first. Before dissection of the para-RLN lymph nodes, an IU probe was inserted through a 10-mm port at the seventh or eighth intercostal space on the mid-axillary line to scan the RLN region (Fig. 1).

**Fig. 2:**
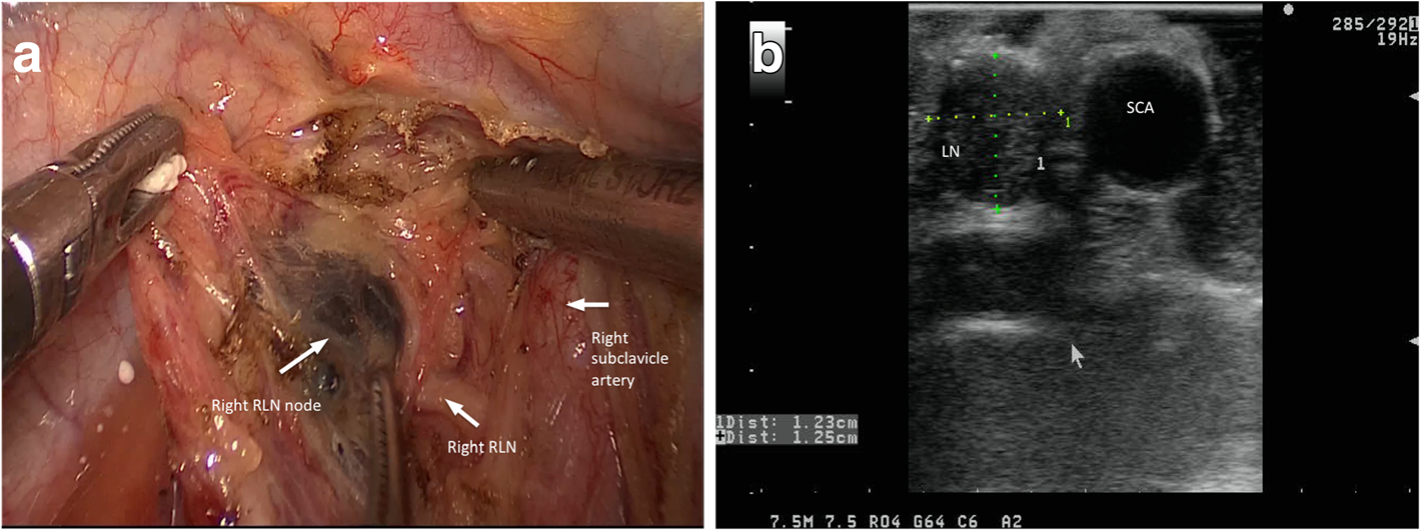
**a** Right thoracic recurrent laryngeal nerve (RLN) node dissection. **b** Intraoperative ultrasound image showed the metastatic lymph node 1.25 cm in diameter located in the right RLN region. SCA, subclavicular artery

**Fig. 3:**
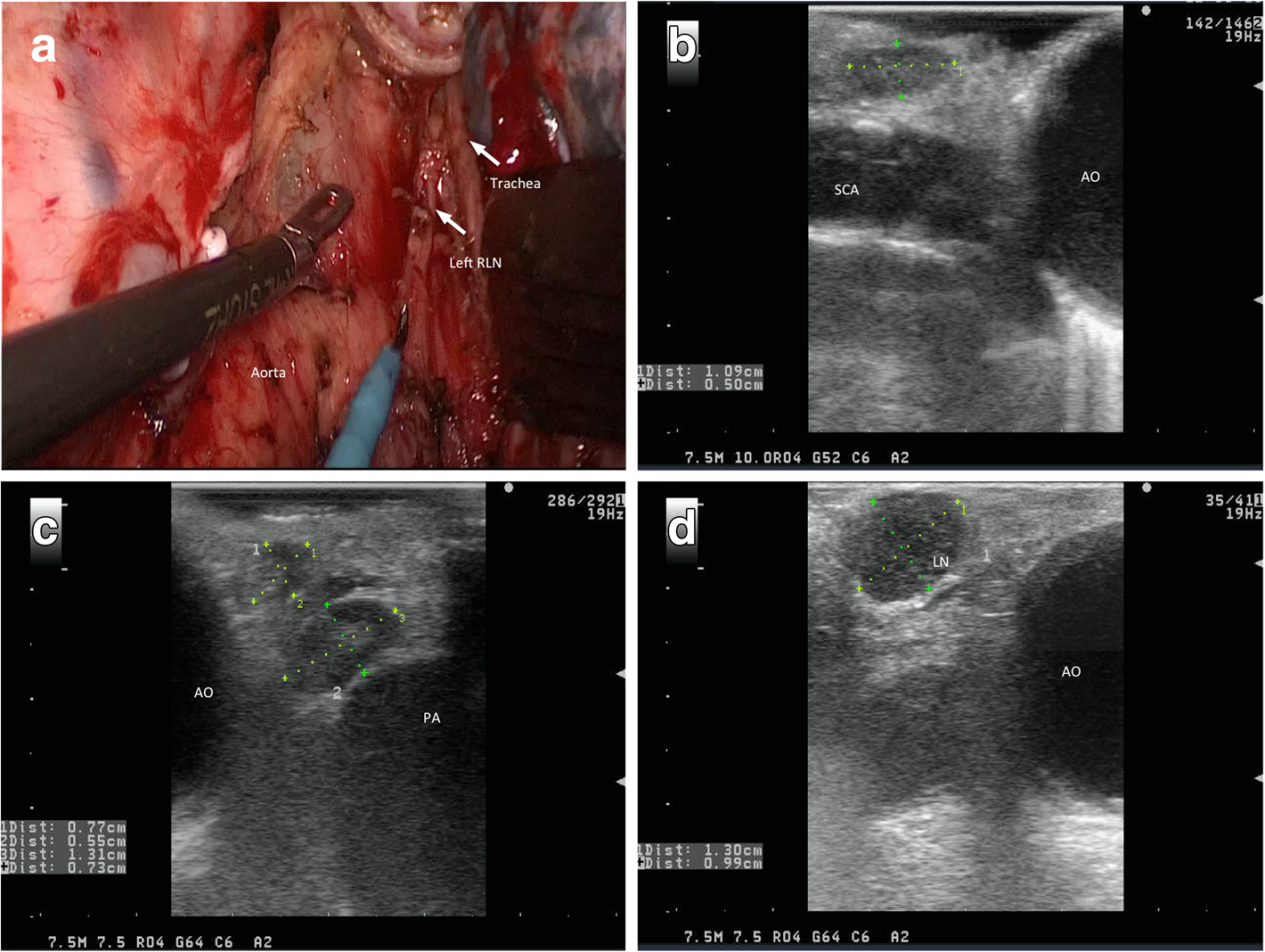
**a** Completed left thoracic recurrent laryngeal nerve (RLN) node dissection. **b** Intraoperative ultrasound image showed one non-metastatic lymph node 0.50 cm in diameter located in the left RLN region. **c** Intraoperative ultrasound image showed the non-metastatic lymph node located in the left RLN region. **d** Intraoperative ultrasound image showed the metastatic lymph node located in the left RLN region. LN, lymph node; AO, aorta; PA, pulmonary artery; SCA, subclavicular artery

The details of the procedure are described in a previous report [5]. Firstly, the right RLN region was detected between esophagus and the right SCA (subclavicular artery) (Fig. 2a). This region was defined through the right SCA, which could be visualised on IU (Fig. 2b). Secondly, the left RLN area was detected by moving the probe to the paratracheal parenchyma in the left (Fig. 3a). The azygos vein was divided routinely. Using a retractor to press the trachea anteriorly, the soft tissue near the left thoracic RLN was scanned by IU. The left RLN region was identified through the aortic arch, the left SCA, the CCA (common carotid artery) and the pulmonary artery in the same side. These arteries were visualised on IU (Fig. 3b–d). By IU, the image of the RLN node was a hypoechoic and round structure which located in the areas described above. Finally, the image characteristics of the lymph node were assessed, including the shortest diameter, S/L ratio, margin and internal echo pattern. The longest diameter was considered as the maximum diameter in longitudinal plane, while the shortest diameter was concerned as the minimum diameter in transverse plane. The size was assessed when the shortest diameter was measured. After measuring the longest and shortest diameters, the S/L ratio could be calculated. The margins were also evaluated, and the lymph nodes were divided into regular and irregular margin type. The features and the locations of these lymph nodes were dicribed on an anatomic figure.

The malignant characteristics of lymph nodes were hypoechogenicity, loss of hilum, S/L > 1, and, irregular margins. The lymph node was diagnosed as malignant as long as one of the features was observed in IU scanning. Each lymph node detected by IU was numbered base on its anatomical location. The same ultrasound specialist, who did not get any information about the results of the CT and EUS, carried out all IU examinations.

After the IU examination, the thoracic RLN node dissection was performed [5]. After resection, the lymph nodes were collected and marked to achcieve an individual node-to-node comparison with the pathologic results. Based on the anatomical position, the node diameter measured, and the shape of the lymph node, individual recognition was confirmed. The lymph node could be repeatedly scanned in saline by the IU probe, if necessary. The pathologists performed the microscopic diagnosis for nodal metastases.

### EUS examination

A conventional endoscopy was performed first in all the cases. EUS was carried out using a 7.5 MHz probe. The probe was covered with a water-filled balloon to realize good transmission of the ultrasound images. The specialist routinely scanned celiac lymph nodes, gastric/esophageal regional lymph nodes, and the invasion depths of the esophageal neoplasm. A lymph node was considered metastatic when it appeared hypoechogenic, roundish, and well demarcated, loss of hilar, larger than 1 cm [6].

### CT examination

All patients underwent a CT scan of the chest and upper abdomen with intravenous contrast. A CT imaging diagnosis of esophageal regional lymph node metastasis was made when the short-axis of the involved lymph node was > 1 cm.

### Data and statistical analysis

The detection of RLN lymph node metastasis in each patient using IU, EUS and CT was compared with pathological examination. The sensitivity, specificity, positive predictive value (PPV), negative predictive value (NPV) and diagnostic accuracy were calculated. The results obtained for each individual node from IU were also compared with the pathological findings. Statistical analysis was performed using SPSS 13.0 for Windows (SPSS Inc., Chicago, IL). Numerical data were expressed as mean ± standard deviation. Categorical data were expressed as counts and proportions. Comparisons were made using unpaired *t*-tests for the means of normally distributed continuous variables. All statistical tests were two-sided. A *P*-value <.05 was considered statistically significant.

According to the International Union Against Cancer and American Joint Committee on Cancer staging system for Esophagus and Esophagogastric Junction, the right and left RLN nodes belong to different node stations with specific anatomic definition. We analyzed the right and left RLN nodes separately [7].

## Results

From January 2014 to June 2016, Sixty patients (51 males and nine females; median age: 60 years) were enrolled at Sun Yat-sen University Cancer Center. The patients’ characteristics are shown in Table 1. All patients achieved complete resection. The most common tumor location was the middle thoracic esophagus. IU scanning took approximately 20 min and had no adverse effect on any patient.

**Table 1:** Patient demographics and tumor characteristics

	Number of patients
Median age, year (range)	60 (46–81)
Gender (%)	
Male	51 (85%)
Female	9 (15%)
Tumor location (%)	
Upper	8 (13.3%)
Middle	44 (73.4%)
Lower	5 (8.3%)
Mutiple	3 (5%)
Clinical stage (%)	
IA	1 (1.7%)
IB	3 (5%)
IIA	1 (1.7%)
IIB	23 (38.3%)
IIIA	20 (33.3%)
IIIB	5 (8.3%)
IIIC	7 (11.7%)
Pathological stage (%)	
IA	2 (3.3%)
IB	7 (11.7%)
IIA	1 (1.7%)
IIB	13 (21.7%)
IIIA	12 (20.0%)
IIIB	10 (16.7%)
IIIC	10 (16.7%)
PCR	5 (8.3%)
Histologic grade (%)	
G1	8 (13.3%)
G2	34 (56.7%)
G3	17 (28.3%)
G4	1 (1.7%)
Operation approach (%)	
Thoracotomy	25 (41.7%)
VATS	35 (58.3%)
Neoadjuvant	
No	46 (76.7%)
CRT	11 (18.3%)
CT	3 (5%)
Intraoperative RLN injury (%)	
Yes	24 (40%)
No	36 (60%)

*Abbreviations*: *CRT* Chemoradiotherapy, *CT* Computed tomography, *PCR* Pathologic complete response, *RLN* Recurrent laryngeal nerve, *VATS* Video-assisted thoracic surgery

All the patients underwent CT and IU examination. EUS could not be performed in 10 patients due to severe esophageal stenosis (7 cases) and suspicious perforation of tumor (3 cases). The sensitivities, specificities, PPVs, NPVs and accuracy by CT, EUS and IU in the diagnosis of right RLN metastasis are presented in Table 2. The sensitivities of IU, CT and EUS for the diagnosis of right RLN node were 71.4, 14.3 and 30.0%, respectively. There was a significant difference in the diagnostic sensitivities of these examinations (χ2 = 10.077, *P* = .006) which indicated that IU had greater sensitivity than CT and EUS. According to the 95% confidence intervals (CIs), there was no significant difference in diagnostic sensitivity for detecting right RLN nodal metastases by either CT or EUS.

**Table 2:** Sensitivities, specificities, positive predictive values, negative predictive values and diagnostic accuracy of intraoperative ultrasonography, computed tomography and endoscopic ultrasonography in the detection of right recurrent laryngeal nerve lymph node metastasis

		Pathology		Sensitivity	Specificity	NPV	PPV	Accuracy
		+	–					
IU	+	10	15	71.4%	67.4%	88.57%	40.00%	68.3%
	–	4	31	95%CI = 41.9%~ 91.6%	95%CI = 51.1%~ 80.0%			
CT	+	2	1	14.3%	97.8%	78.95%	66.67%	78.3%
	–	12	45	95%CI = 51.1%~ 80.0%	95%CI = 82.8%~ 99.9%			
EUS	+	3	2	30.0%	95.0%	84.44%	60.00%	82%
	–	7	38	95%CI = 6.7%~ 65.2%	95%CI = 83.8%~ 99.4%			

Abbreviations: *CI* Confidence interval, *CT* Computed tomography, *EUS* Endoscopic ultrasonography, *IU* Intraoperative ultrasonography, *NPV* Negative predictive value, *PPV* Positive predictive value, *RLN* Recurrent laryngeal nerve

For diagnosis of right RLN lymph nodes. The sensitivity had statistically significant difference (χ2 = 10.077, *P* = 0.006)

For diagnosis of right RLN lymph nodes. The specificity had statistically significant difference. (χ2 = 21.725, *P* < 0.001)

For diagnosis of right RLN lymph nodes. The NPV had no statistically significant difference (χ2 = 1.511, *P* = 0.470)

For diagnosis of right RLN lymph nodes. The PPV had no statistically significant difference (χ2 = 1.271, *P* = 0.530)

For diagnosis of right RLN lymph nodes. The diagnostic accuracy had no statistically significant difference(χ2 = 3.088, P = 0.214)

The diagnostic specificities of IU, CT and EUS for detection of right RLN node metastases were 67.4, 97.8 and 95.0%, respectively. There was a significant difference in these diagnostic specificities (χ2 = 21.725, *P* < .001). The PPVs and NPVs of these examinations are shown in Table 2. No significant difference was observed in either PPV or NPV.

The accuracy of the diagnosis of right RLN lymph nodes metastases by IU, EUS and CT is shown in Table 2. No significant difference was observed in the accuracy (χ2 = 3.088, *P* = 0.214).

Table 3 shows the sensitivities, specificities, PPVs, NPVs and accuracy for the diagnosis of left RLN metastasis by IU, CT and EUS. The diagnostic sensitivities of IU, CT and EUS were 91.7, 16.7 and 40.0%, respectively. There was a significant difference in these diagnostic sensitivities (χ2 = 14.067, *P* = .001). The diagnostic sensitivity of IU for detection of left RLN lymph node metastases was significantly higher than EUS or CT. Additionally, no significant difference was observed in the diagnostic sensitivity of either EUS or CT for detecting RLN metastases.

**Table 3:** Sensitivities, specificities, positive predictive values, negative predictive values and diagnostic accuracy of intraoperative ultrasonography, computed tomography, and endoscopic ultrasonography in the detection of left recurrent laryngeal nerve lymph node metastasis

		Pathology		Sensitivity	Specificity	NPV	PPV	Accuracy
		+	–					
IU	+	11	10	91.7%	79.2%	97.44%	52.38%	81.7%
	–	1	38	95%CI = 61.4%~ 99.8%	95%CI = 66.2%~ 90.0%			
CT	+	2	0	16.7%	100.0%	82.76%	100%	83.3%
	–	10	48	95%CI = 2.1%~ 43.4%	95%CI = 92.6%~ 100.0%			
EUS	+	4	7	40.0%	82.5%,	84.62%	36.36%	74%
	–	6	33	95%CI = 12.2%~ 76.6%	95%CI = 66.9%~ 97.4%			

Abbreviations: *CI* Confidence interval, *CT* Computed tomography, *EUS* Endoscopic ultrasonography, *IU* Intraoperative ultrasonography, *NPV* Negative predictive value, *PPV* Positive predictive value, *RLN* Recurrent laryngeal nerve

For diagnosis of left RLN lymph nodes. The sensitivity had statistically significant difference (χ2 = 14.067, *P* = 0.001)

For diagnosis of left RLN lymph nodes. The specificity had statistically significant difference. (χ2 = 10.819, *P* = 0.004)

For diagnosis of left RLN lymph nodes. The NPV had no statistically significant difference (χ2 = 5.009, *P* = 0.82)

For diagnosis of left RLN lymph nodes. The PPV had no statistically significant difference (χ2 = 2.866, *P* = 0.239)

For diagnosis of left RLN lymph nodes. The diagnostic accuracy had no statistically significant difference (χ2 = 1.646, P = 0.439)

The diagnostic specificities of IU, CT and EUS for detection of left RLN node metastases were 79.2, 100 and 82.5%, respectively. There was a significant difference in the diagnostic specificities (χ2 = 10.819, *P* = .004). In addition, no significant difference was observed in either PPV or NPV of these examinations in diagnosing left RLN node metastasis.

The accuracy of the diagnosis of left RLN lymph nodes metastases by EUS, CT and IU showed no significant difference (χ2 = 1.646,*P* = 0.439), the results are shown in Table 3.

## Discussion

Total dissection of mediastinal lymph nodes is very important for the radical resection of patients with esophageal squamous cell carcinoma. The dissection of RLN lymph nodes is, therefore, a key component and challenge. Previous studies [8–12] have shown esophageal cancer patients had a high incidence of RLN lymph nodal metastasis with the range from 20 to 30%.

Tan et al. [13] reported that RLN lymphadenectomy could decrease local recurrence, and significantly prolong the overall survival of patients with esophageal cancer. However, the dissection of RLN nodes caused a 14–30% increase in RLN injury [2, 14–16]. RLN injury can lead to a significant increase in cardiopulmonary complications and anastomosis leakage, as well as a decreased long-term quality of life [2–4]. Theoretically, accurate preoperative assessment of the RLN lymph nodes would be helpful to perform selective RLN lymph node dissection, in order to reduce RLN injury.

Nevertheless, the accuracy of routine examinations in the detection of RLN node metastases is far from optimal. Our study showed that IU had significantly higher sensitivity for the detection of RLN node metastases, compared with CT or EUS.

For esophageal cancer, CT is crucial in detecting primary tumor, distant metastases, and regional nodal involvement. However, one of the major problems with the use of CT in the staging of esophageal cancer is the inability to identify small lymph node metastases, which may lead to under-staging.

On CT, lymph nodes > 1 cm in short axis diameter are considered abnormal. However, CT can supply little information about the detailed structure of the diseased lymph node [17]. Another major shortcoming of CT is that its standard for detecting metastases, i.e., the shortest diameter of a lymph node should be > 1.0 cm, supplies little information regarding the content of the lymph node. For upper mediastinal lymph nodes in particular, this standard is unsuitable as most positive upper mediastinal nodes have diameters which range from 4 to 8 mm.

In the current study, both specificity and PPV of CT were highest. If RLN node dissection had been waived when no metastatic RLN nodes were detected by CT, most patients without metastasis would have avoided RLN lymphadenectomy. However, over 50% of the patients with RLN node metastasis would have lost necessary RLN lymphadenectomy and the opportunity of radical resection. Therefore, CT is not a practical approach for selective RLN lymphadenectomy.

The superiority of EUS for assessing both T and N stage disease has been confirmed in several studies [18–20]. However, our results showed that the sensitivity of EUS for detection of RLN lymph nodes was no higher than 40%, which was consistent with a previous study [21]. This finding may have resulted because the endoscopic ultrasound was attenuated and scattered by air-containing obstacles such as the trachea and lungs. If selective RLN lymphadenectomy had been performed based on EUS, nodal metastases would have been missed in more than half of the patients in which nodal metastases were present.

In our study, IU had a significantly higher sensitivity compared with either CT or EUS. Moreover, the NPV of IU for the detection of RLN nodes was more than 88%.

IU has some obvious advantages. Firstly, compared with CT, IU detects more information on the innate character of lymph nodes, including the size, shape, and internal echogenicity. Secondly, the IU probe can directly and thoroughly scan the superior mediastinum the parenchyma. As a result, IU overcomes interference from air-containing obstacles and obtains good-quality images. Lastly, IU can evaluate the RLN nodes one by one. Through matching the result of each individual RLN node between IU and the pathological examination, diagnostic criteria could be improved. However, for the right RLN node, the IU sensitivity of 71.4% was unsatisfactory. This was a major limitation of our study because for some patients, the IU probe pushed the right RLN nodes into the neck and the IU operator could not scan the nodes and missed them. This factor seldom affected the detection of left RLN nodes, so the sensitivity of IU was 91.7% for the left RLN nodes. In order to overcome this problem, the IU probe needs to be improved.

## Conclusions

IU showed superior sensitivity compared with preoperative CT or EUS in detecting RLN lymph node metastasis in patients with thoracic esophageal cancer. More studies are needed to further assess the utility of IU for selective RLN lymphadenectomy.

## Abbreviations

AO: Aorta
CCA: Common carotid artery
CI: COnfidence interval
CT: Computed tomography
EUS: Endoscopic ultrasonography
IU: Intraoperative ultrasonography
NPV: Negative predictive value
PA: Pulmonary artery
PPV: Positive predictive value
RLN: Recurrent laryngeal nerve
SCA: Subclavicular artery
VATS: Video-assisted thoracic surgery
